# Supplementary material for: Identification and functional analysis of non-coding regulatory small RNA FenSr3 in Bacillus amyloliquefaciens LPB-18
Source: PeerJ. 2023 May 15;11:e15236. doi: 10.7717/peerj.15236 (PMC10194069; doi:10.7717/peerj.15236)
Supplement: Supplemental Information 4 [file peerj-11-15236-s004.zip › KO/CK-vs-T1_map/map03050.html]

KEGG PATHWAY: Proteasome - Reference pathway


|  |  |
| --- | --- |
| **Proteasome - Reference pathway** |  |

[
Pathway menu
| Organism menu
| Pathway entry
| Show description
| User data mapping
]

The proteasome is a protein-destroying apparatus involved in many essential cellular functions, such as regulation of cell cycle, cell differentiation, signal transduction pathways, antigen processing for appropriate immune responses, stress signaling, inflammatory responses, and apoptosis. It is capable of degrading a variety of cellular proteins in a rapid and timely fashion and most substrate proteins are modified by ubiquitin before their degradation by the proteasome. The proteasome is a large protein complex consisting of a proteolytic core called the 20S particle and ancillary factors that regulate its activity in various ways. The most common form is the 26S proteasome containing one 20S core particle and two 19S regulatory particles that enable the proteasome to degrade ubiquitinated proteins by an ATP-dependent mechanism. Another form is the immunoproteasome containing two 11S regulatory particles, PA28 alpha and PA28 beta, which are induced by interferon gamma under the conditions of intensified immune response. Other regulatory particles include PA28 gamma and PA200. Although PA28 gamma also belongs to a family of activators of the 20S proteasome, it is localized within the nucleus and forms a homoheptamer. PA28 gamma has been implicated in the regulation of cell cycle progression and apoptosis. PA200 has been identified as a large nuclear protein that stimulates proteasomal hydrolysis of peptides.

|  |  |  |
| --- | --- | --- |
| Reference pathway | 184% 150% 122% 100% 82% 67% 55% | 图片下载 |
